# Advancing provitamin A biofortification in sorghum: from allele mining and marker development to prebreeding and breeding

**DOI:** 10.1007/s00122-026-05245-2

**Published:** 2026-04-27

**Authors:** Clara Cruet-Burgos, Linly Banda, Jacques M. Faye, Cyril Diatta, David Zapata, Geoffrey P. Morris, Davina H. Rhodes

**Affiliations:** 1 Department of Horticulture & Landscape Architecture, Colorado State University, Fort Collins, Colorado 80523 USA; 2 Department of Soil and Crop Science, Colorado State University, Fort Collins, Colorado 80523 USA; 3 Institut Sénégalais de Recherches Agricoles, Centre d’Étude Régional Pour L’Amélioration de L’Adaptation À La Sécheresse, Centro Internacional de Mejoramiento de Maíz y Trigo (CIMMYT), Thiès, Sénégal; 4 Institut Sénégalais de Recherches Agricoles, Centre d’Étude Régional Pour L’Amélioration de L’Adaptation À La Sécheresse, Centre National de Recherches Agronomiques, Bambey, Sénégal

## Abstract

**Key message:**

Allele mining for provitamin A biofortification in sorghum is advanced with new pre-bred germplasm and markers that are trait-predictive in diverse germplasm and breeding programs.

**Abstract:**

Provitamin A biofortification of the staple cereal crop sorghum (*Sorghum bicolor* [L.] Moench) could reduce vitamin A deficiency in low-income countries of sub-Saharan Africa. To advance mining of alleles that confer high provitamin A from genebank accessions, we developed prebreeding germplasm and trait-predictive markers, and established the utility of these markers in a range of prebreeding and breeding materials. We first tested the hypothesis that carotenoid concentrations in sorghum grain can be increased through breeding using six biparental families developed from high-carotenoid genebank accessions. The families exhibited abundant transgressive segregation for carotenoid concentrations, with positive asymmetry in many cases (4 of 6 families) suggesting the release of epistatic variation. Next, we developed eleven markers from SNPs associated with carotenoid variation, and validated their predictiveness in the six biparental families and diverse global germplasm. Two markers (snpSB00267 and snpSB00276), previously identified as zeaxanthin QTLs, were predictive of provitamin A content (β-carotene) in the biparental family evaluated and the diverse germplasm, and require further validation. Five markers that were predictive of zeaxanthin content—most notably snpSB00265, located within the *zeaxanthin epoxidase* (*ZEP*) gene; snpSB00277 and snpSB00264, near *ZEP*; and snpSB00279 and snpSB00280, in the *β-carotene 3-hydroxylase* gene—are recommended for indirect selection for provitamin A carotenoids. In a Senegalese breeding program, most markers (9 of 11) were segregating and contrasted for the elite germplasm (low carotenoid alleles) and yellow-endosperm donor lines (high carotenoid). Together, findings show that sorghum grain carotenoids can be increased through breeding, and demonstrate the potential of marker-assisted selection for provitamin A biofortification.

**Supplementary Information:**

The online version contains supplementary material available at 10.1007/s00122-026-05245-2.

## Introduction

Vitamin A deficiency is a major global public health issue, particularly in low-income countries where starchy staple crops such as cereals and tubers are the main source of calories (World Health Organization 2009; Bailey et al. 2015; Stevens et al. 2015; Wirth et al. 2017; Song et al. 2023). In sub-Saharan Africa, nearly half the population is affected, leading to decreased immune function, blindness, and death (Stevens et al. 2015; Song et al. 2023). In these regions, dietary vitamin A is derived predominantly from plant carotenoids—the yellow, orange, and red colored pigments in plants—with α-carotene, β-carotene, and β-cryptoxanthin serving as the primary provitamin A compounds that the body converts to vitamin A after consumption. Of these provitamin A carotenoids, β-carotene is the most abundant and nutritionally significant because each β-carotene molecule can be enzymatically cleaved to yield two molecules of vitamin A (retinol), while α-carotene and β-cryptoxanthin yield only one. However, cereals and tubers generally accumulate only low concentrations of these provitamin A carotenoids. As a result, biofortification of crops such as maize (*Zea mays*), cassava (*Manihot esculenta*), and sweetpotato (*Ipomoea batatas*) has emerged as a sustainable strategy to reduce vitamin A deficiencies (Andersson et al. 2017), with multiple studies demonstrating its efficacy in improving the nutritional status of communities with high rates of micronutrient deficiencies (Hotz et al. 2012; Gannon et al. 2014; Palmer et al. 2016, 2018; Low et al. 2017).

Sorghum (*Sorghum bicolor* [L.] Moench) is a major staple crop in drought-prone regions of sub-Saharan Africa where vitamin A deficiencies are prevalent (Stevens et al. 2015; Song et al. 2023, 2025), making it a promising target for vitamin A biofortification (Worzella et al. 1965; Fernandez et al. 2008; Cruet‐Burgos et al. 2020a; Cruet-Burgos et al. 2023; McDowell et al. 2024). Biofortified sorghum would complement other biofortification efforts by providing a reliable source of vitamin A in drought-prone regions where it remains a dietary staple when other crops fail. Despite this potential, most sorghum varieties predominantly accumulate non-provitamin A carotenoids, particularly lutein and zeaxanthin, with concentrations ranging between 0.02–9.4 µg/g and 0.01–9.1 µg/g, respectively, and low concentrations of provitamin A carotenoids (Blessin et al. 1962; Worzella et al. 1965; Fernandez et al. 2008; Cardoso et al. 2015; Shen et al. 2017; Cruet‐Burgos et al. 2020), mainly in the form of β-carotene, typically ranging between 0.07–0.80 µg/g (Cruet-Burgos et al. 2023). However, genetic variation exists, with yellow endosperm sorghums showing relatively higher carotenoid concentrations (Fernandez et al. 2008; McDowell et al. 2024). Although yellow endosperm sorghum has been reported in Ethiopia, Nigeria, and a few other regions (McDowell et al. 2024), they are not widely cultivated and occur mainly as landraces. In contrast, most improved and farmer-preferred varieties in Africa are non-yellow, and therefore remain low in carotenoids (Reddy et al. 2008; Faye et al. 2022). Based on average sorghum consumption patterns across the target regions (FAO.FAOSTAT 2025) together with estimates of processing degradation and nutrient bioavailability (Li et al. 2010; Kean et al. 2011), we estimated an initial biofortification target value of 12 μg/g β-carotene to provide 25% of the estimated average requirement (EAR) for vitamin A.

Therefore, breeding is needed to transfer favorable carotenoid alleles from landraces into elite, farmer-preferred backgrounds. Global genebanks, which hold over 150,000 sorghum accessions (Genesys Team 2025), harbor tremendous genetic diversity for mining favorable alleles (Cruet‐Burgos et al. 2020a). Early work demonstrated that provitamin A concentrations (β-carotene) can respond to selection (Worzella et al. 1965), and more recent studies identified genebank accessions with relatively high grain carotenoids (Cruet‐Burgos et al. 2020; Cruet-Burgos et al. 2023). However, efforts to breed for increased carotenoid content is constrained by several factors: (i) limited access to elite donor lines with known carotenoid levels, which complicates parent selection for crossing; (ii) reliance on non-elite donors, which necessitates advancement of all progeny to physiological maturity before nutrient evaluation, delaying selection and requiring substantial field space; and (iii) moderate carotenoid heritability, which requires evaluation of large populations over multiple cycles to reliably identify and fix high-carotenoid lines. These biological and logistical constraints are compounded by the demands of nutrient phenotyping, which is time-consuming, labor-intensive, and highly technical (Araus and Cairns 2014; Helguera et al. 2020). The most accurate quantification of carotenoids is via high-performance liquid chromatography (HPLC), which requires specialized equipment, extensive user training, and expensive consumables (Rivera and Canela-Garayoa 2012; Amorim-Carrilho et al. 2014). Biofortification breeding requires phenotyping of thousands of progeny, so HPLC is generally not feasible for low-resourced programs in developing countries (Rosales et al. 2022).

Carotenoid biofortification efforts primarily use indirect selection methods, such as grain color or marker-assisted selection (MAS), due to the challenge of scalable phenotypic selection (Chandler et al. 2013; Zunjare et al. 2018). In sorghum, color-based phenotyping is an effective qualitative approach for total carotenoids, but not effective for quantitatively selecting or distinguishing between provitamin A and non-provitamin A carotenoids (Fernandez et al. 2008; McDowell et al. 2024). MAS is effective for oligogenic traits (conditioned by only a few genes) (Hasan et al. 2021), such as carotenoid concentration (Cruet‐Burgos et al. 2020a). In maize, MAS targeting carotenoid biosynthesis genes *β-carotene hydroxylase 1* and *lycopene epsilon cyclase* has been successful (Zunjare et al. 2018). MAS requires breeder-friendly markers that are (i) causal variants or co-segregate with the causal variant, (ii) trait-predictive for selection, (iii) polymorphic in target germplasm, and (iv) cost-effective relative to the breeding program’s budget (Mohler and Singrün 2005; Hasan et al. 2021). Kompetitive allele specific PCR (KASP) markers are particularly useful for breeding programs in low-income countries lacking well-resourced lab facilities, due to their high throughput and availability through outsourced platforms such as Intertek, the CGIAR Excellence in Breeding platform, and LGC Genomics, at a low cost of less than US$5 per assay (Intertek) (Semagn et al. 2014; Excellence In Breeding 2023). Another advantage of KASP markers is that they can be applied as early as the first segregating generation (F_2_), enabling efficient selection and minimizing the phenotyping requirements in subsequent generations.

**Fig. 1:**
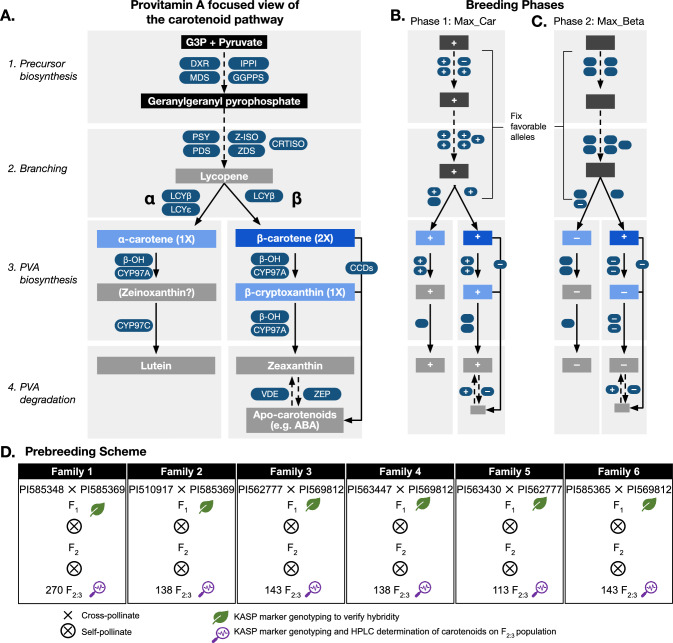
Molecular breeding for provitamin A biofortification in sorghum. **A** Putative biochemical pathways for grain carotenoids in sorghum. Compounds are shown in rectangles, with precursors of carotenoids denoted in black, provitamin A (PVA) carotenoids denoted in blue, and non-PVA carotenoids and apo-carotenoids in gray. Enzymes are denoted with ovals (*β–OH*, β-carotene hydroxylase; CCD, carotenoid cleavage dioxygenase; CRTISO, carotenoid isomerase; CYP97A, cytochrome P450 97A; CYP97C, cytochrome P450 97C; DMAPP, dimethylallyl diphosphate; DXR, 1-deoxy-d-xylulose 5-phosphate reductoisomerase; GGPPS, geranylgeranyl diphosphate synthase; IPPI, isopentenyl diphosphate isomerase; LCYβ, lycopene β-cyclase; LCYε, lycopene ε-cyclase; MDS, ME-cPP synthase; PDS, phytoene desaturase; PSY, phytoene synthase; VDE, violaxanthin de-epoxidase; ZDS, δ-carotene desaturase; ZEP, zeaxanthin epoxidase; Z-ISO, 15-cis-δ-carotene isomerase). The number of retinol molecules cleaved from each PVA compound (1 × or 2 ×) is noted. Two possible biofortification phases are shown schematically (with the structure of each diagram corresponding to the pathway in panel A): **B** maximizing carotenoids (Max_Car) and **C** maximizing β-carotene (Max_Beta). Positive signs indicate where high-activity alleles would be selected using phenotypic selection (rectangle) or genotypic selection (ovals), while negative signs indicate where low-activity alleles would be selected. **D** Prebreeding scheme and experimental workflow for this study, including biparental families development, KASP marker genotyping, and carotenoid phenotyping. F_1_ plants were genotyped with KASP markers to confirm hybridity. F_2_ plants were genotyped with KASP markers and F_2:3_ grain was phenotyped for carotenoids through HPLC quantitation to determine the marker-trait association

**Fig. 2:**
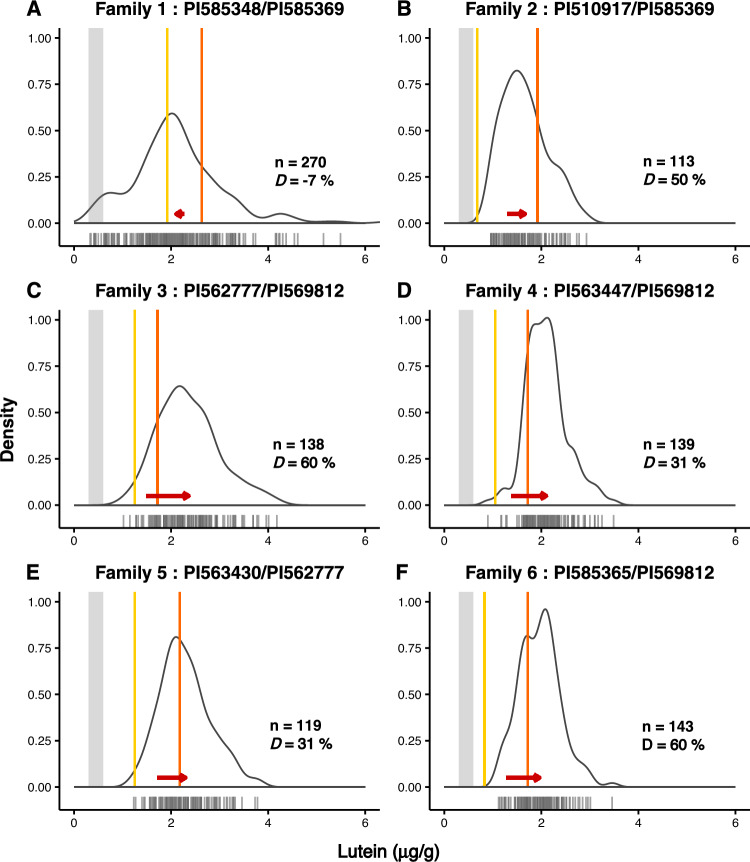
Symmetrical and asymmetrical transgressive segregation of lutein, a non-provitamin A degradation product of provitamin A carotenoids on the α branch. Distribution of lutein amongst the six unselected F_2:3_ families and their parents (where ‘n’ denotes the number of progeny per family and* D* represents the percentage increase over the midparent value). **A** Family 1 and their parents PI585348 and PI585369; **B** Family 2 and their parents PI510917 and PI585369; **C** Family 3 and their parents PI562777 and PI569812; **D** Family 4 and their parents PI563447 and PI569812; **E** Family 5 and their parents PI563430 and PI562777, and **F** Family 6 and their parents PI585365 and PI569812. The vertical lines represent the lutein concentration range (0.3–0.6 µg/g) in elite African germplasm (CSM-63, IRAT204, Macia, and Mota Maradi; grey), the lower-parent concentration (yellow), and the higher-parent concentration (orange). The red arrow represents the direction and magnitude of the difference between the midparent value (tail) and the average value of the progeny (head)

**Fig. 3:**
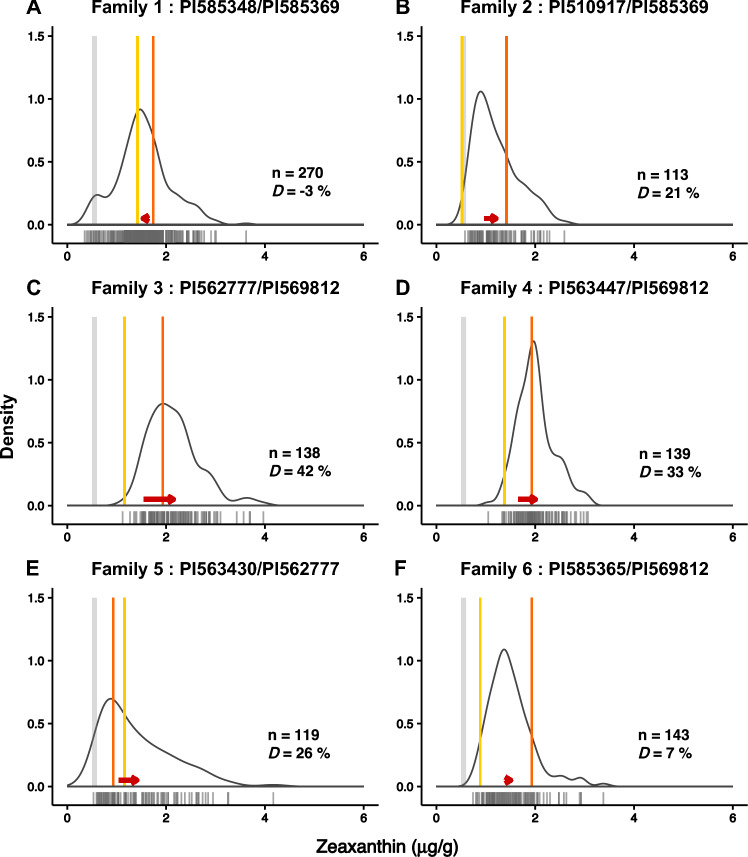
Symmetrical and asymmetrical transgressive segregation of zeaxanthin, a non-provitamin A degradation product of provitamin A carotenoids on the β branch. Distribution of zeaxanthin among the six unselected F_2:3_ families and their parents (where ‘n’ denotes the number of progeny per family and *D* represents the percentage increase over the midparent value). **A** Family 1 and their parents PI585348 and PI585369; **B** Family 2 and their parents PI510917 and PI585369; **C** Family 3 and their parents PI562777 and PI569812; **D** Family 4 and their parents PI563447 and PI569812; **E** Family 5 and their parents PI563430 and PI562777; and (**F**) Family 6 and their parents PI585365 and PI569812. The vertical lines represent the zeaxanthin concentration range (0.5–0.6 µg/g) in elite African germplasm (CSM-63, IRAT204, Macia, and Mota Maradi; grey), the lower-parent concentration (yellow), and the higher-parent concentration (orange). The red arrow represents the direction and magnitude of the difference between the midparent value (tail) and the average value of the progeny (head)

**Fig. 4:**
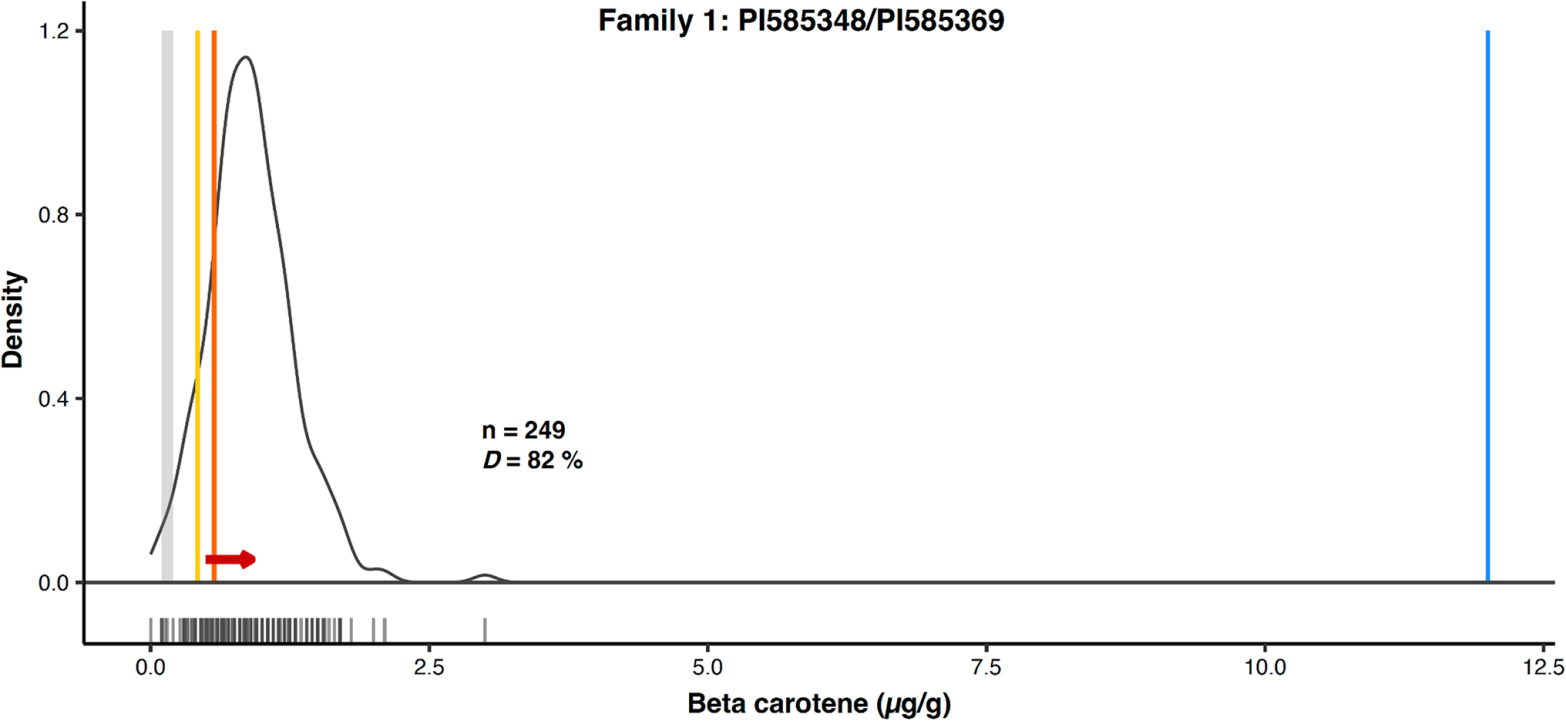
Positive asymmetric transgressive segregation for β-carotene in a high-by-high biparental family. Distribution of β-carotene in family 1 (PI585348 ✕ PI585369), where ‘n’ denotes the number of progeny per family and *D* represents the percentage increase over the midparent value. Vertical lines represent the β-carotene concentration range (0.1–0.2 µg/g) in representative African varieties (CSM-63, IRAT204, Macia, and Mota Maradi; grey), the lower-parent concentration (yellow), higher-parent concentration (orange), and the initial biofortification target concentration (~ 12 µg/g; blue). The red arrow represents the direction and magnitude of the difference between the midparent value (tail) and the mean of the progeny (head). The increase in the β-carotene concentration of the progeny of family 1 compared to both parents and elite germplasm is favorable for biofortification

**Fig. 5:**
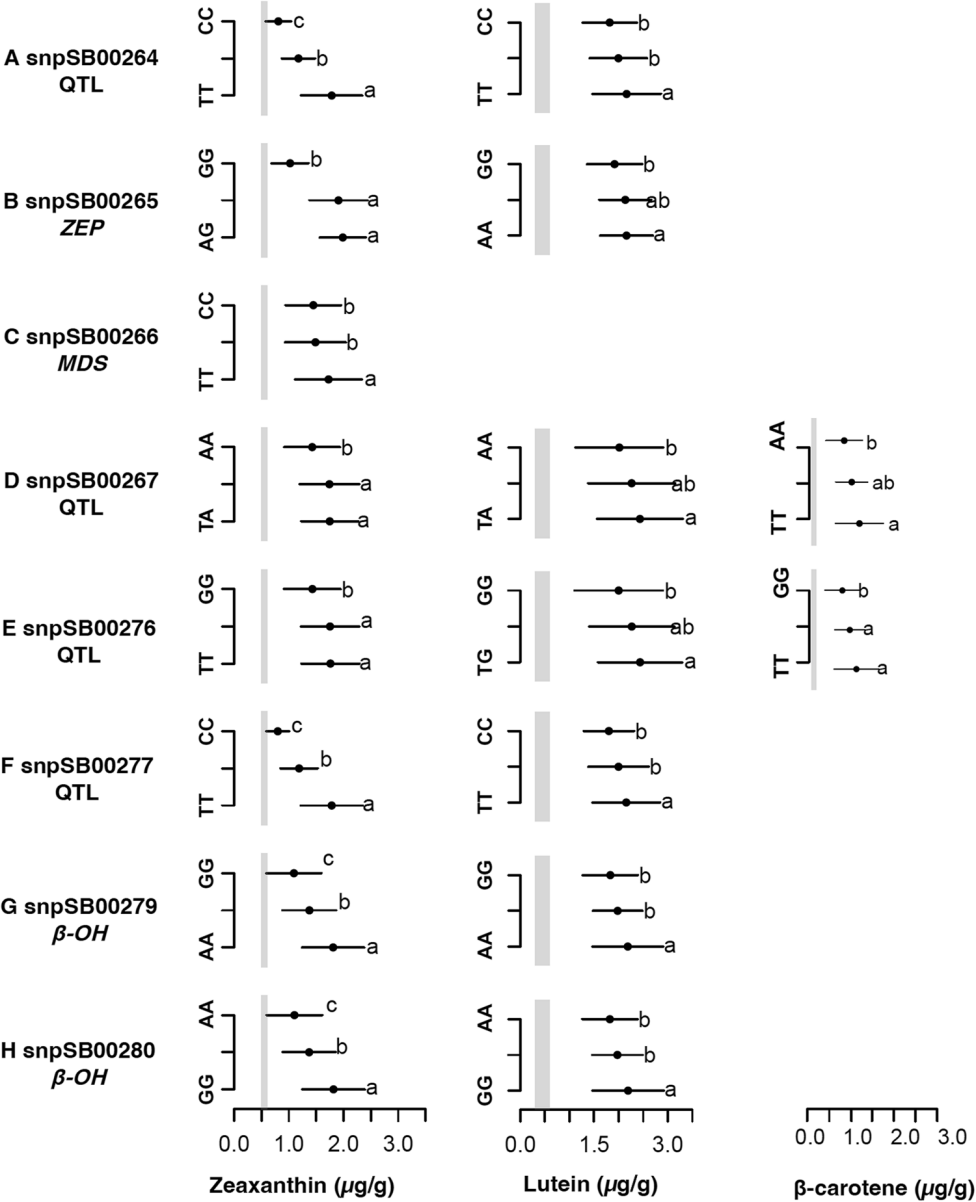
Several KASP markers accurately predict rank of lutein, zeaxanthin, and β-carotene content among genotypic classes in biparental families. Least significant difference (LSD, alpha = 0.05) for markers associated with lutein (*n* = 919), zeaxanthin (*n* = 919), and β-carotene (*n* = 246) concentrations in F_2:3_ progenies. The grey vertical line represents the range of carotenoid concentration (lutein: 0.3–0.6 µg/g, zeaxanthin: 0.5–0.6 µg/g, and β-carotene: 0.1–0.2 µg/g) in representative African varieties (CSM-63, IRAT204, Macia, and Mota Maradi). Eight markers accurately predicted zeaxanthin, seven predicted lutein, and two predicted β-carotene concentration. Only two markers tagging zeaxanthin QTLs (snpSB00267 and snpSB00276) predicted all three carotenoid types while the markers tagging *ZEP* and *β–OH* predicted both lutein and zeaxanthin content

**Fig. 6:**
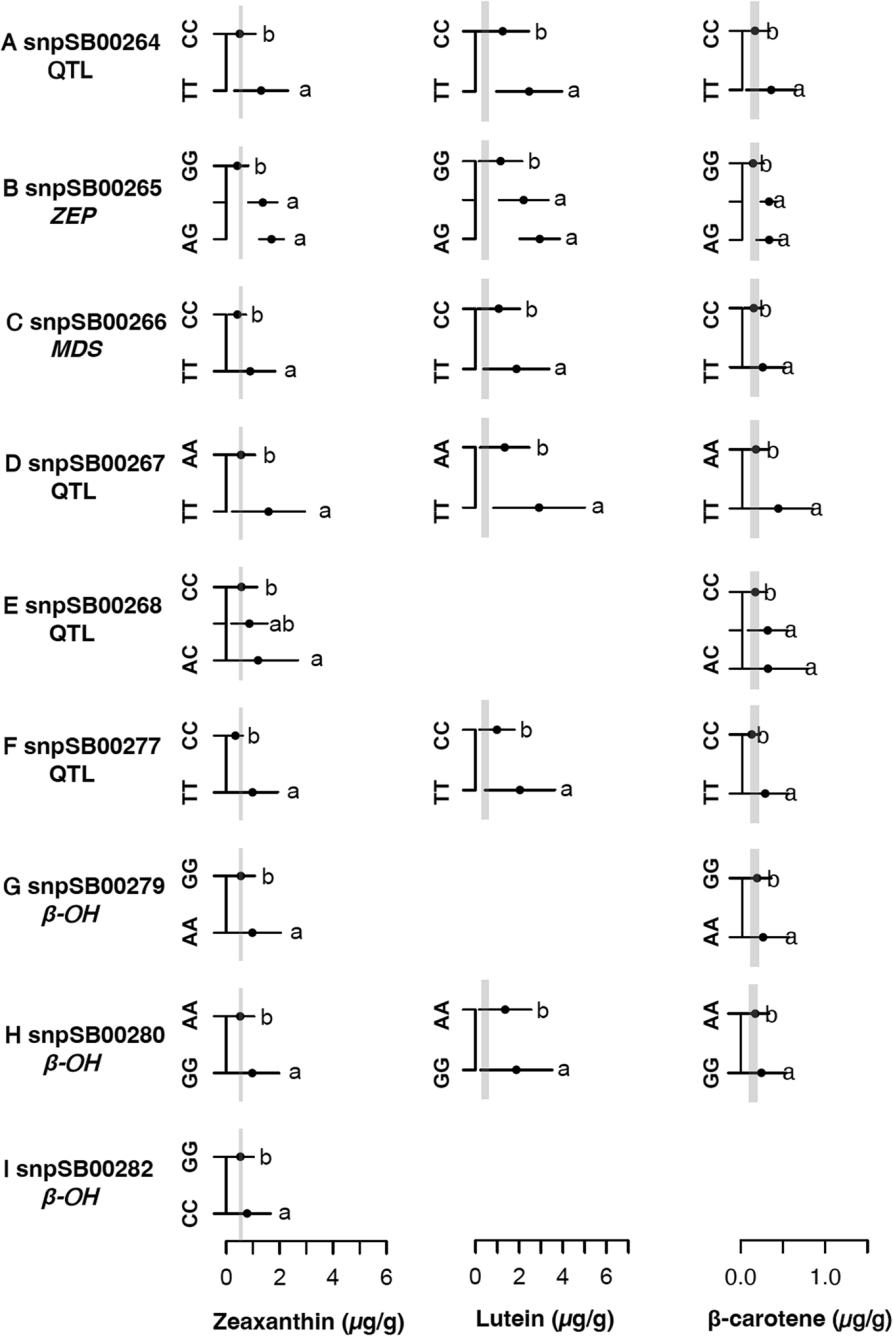
Several KASP markers accurately predict rank of lutein, zeaxanthin, and β-carotene content among genotypic classes in diverse global germplasm. Least significant difference (LSD; alpha = 0.05) for markers associated with lutein, zeaxanthin, and β-carotene concentrations in global genebank accessions (*n* = 90). Grey vertical line represents the range of carotenoid concentration (lutein: 0.3–0.6 µg, zeaxanthin: 0.5–0.6 µg, β-carotene: 0.1–0.2 µg) in four representative African varieties (CSM-63, IRAT204, Macia, and Mota Maradi). Nine markers were trait-predictive for zeaxanthin, six for lutein, and eight for β-carotene

**Fig. 7:**
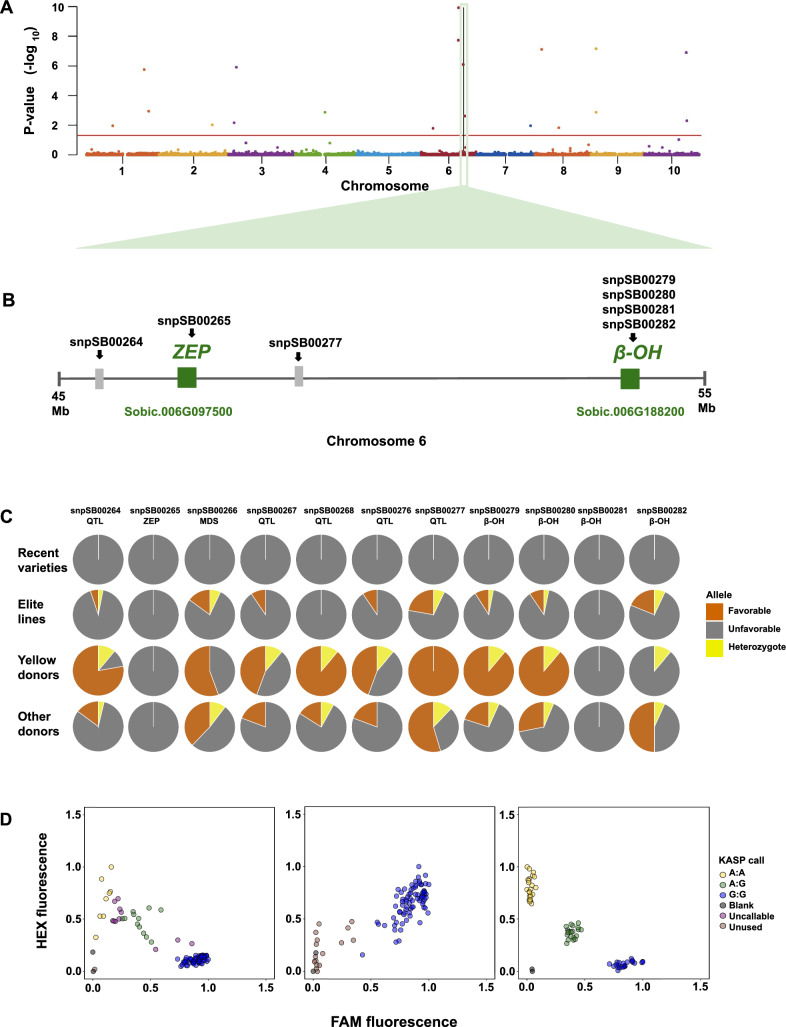
From allele mining to technology deployment in breeding programs. A summary demonstrating the synthesis between previous studies and the current study: A genome-wide association study (GWAS) based on previously published data (Cruet‐Burgos et al. 2020b) identified several marker trait associations (MTAs) for zeaxanthin content, with MTAs in chromosome 6 explaining between 1.3 and 8.9% phenotypic variance. In this study the loci were successfully converted into trait predictive KASP markers that identified segregation in germplasm in the ISRA breeding program. Yellow donor lines had a higher frequency of the favorable alleles for high carotenoid content, which were absent in the recently released varieties. **A** Manhattan plot of association with zeaxanthin content (n = 310). The horizontal red line represents the genome-wide significance threshold at 5% FDR. The vertical black line represents the start coordinates for the *ZEP* gene. **B** A focus on chromosome 6 showing the positions of the markers developed and tested in this study: snpSB00264 (385 kb from *ZEP*), snpSB00265 (located inside the *ZEP* gene, Sobic.006G097500), snpSB00277 (921 kb from *ZEP*), snpSB0079—snpSB00281 (located inside the *β–OH* gene, Sobic.006G188200). The green boxes represent carotenoid genes, the grey boxes represent non-carotenoid genes (not drawn to scale, but representative of relative positions between 45 and 55 Mbs of the chromosome). **C** Frequency of alleles in candidate genes tagged by KASP markers in breeding and prebreeding germplasm consisting of recently released varieties (*n* = 4), elite lines (*n* = 98), selected yellow-grained donor lines (*n* = 9), and other donor lines being used for non-carotenoid traits (*n* = 26). The yellow donor lines have a significantly higher frequency (*p* < 0.05) of favorable alleles across the markers tested, except for snpSB00265 and snpSB00281, which were monomorphic. The favorable alleles for carotenoid content are not present in the recently released ISRA varieties (first row; Nganda, Faourou, Golobe, Payenne) and are uncommon in elite lines from the ISRA program (second row). By contrast, many of the favorable alleles are found at high frequency in the yellow donor lines (third row), and found at moderate frequency in other donor lines being used by the ISRA program (fourth row). **D** Validation of markers across populations revealed that one of our most promising trait-predictive markers (snpSB00265) performed poorly in the diverse germplasm (left), with a number of lines classified as ‘uncallable’- amplified but not assigned to a cluster. Its performance was worse in the ISRA germplasm (middle), where most of the lines hypothesized to have the favorable allele were reported as ‘unused’- unamplified. To address this, primers were redesigned on the antisense strand (marker snpSB00869), and this substantially improved the performance of the marker (right). These findings emphasize the need to validate markers across diverse genetic backgrounds prior to deployment

In this study, we sought to advance an allele mining approach for provitamin A biofortification in sorghum, accounting for the complexities of the carotenoid pathway such as branching structure, multi-functional enzymes, and multiple potential provitamin A targets (Fig. 1A). Given this complexity, we envision that various phenotypic and genotypic selection targets could be combined to maximize total carotenoids in a first phase of biofortification (Max_Car, Fig. 1B). This is based on the observation that the total carotenoid content—mostly composed of the non-provitamin molecules lutein and zeaxanthin—is strongly and positively correlated with β-carotene (0.86–0.94, *p* < 0.001) (McDowell et al. 2024). We therefore hypothesize that increasing total carotenoid content will concomitantly increase β-carotene and possibly other provitamin A carotenoids to some extent; thus, non-provitamin A carotenoids can be used as a proxy for selection. However, this approach alone would be insufficient to reach the target value. The second phase of biofortification would then focus on maximizing specific provitamin A carotenoids, most importantly β-carotene (Max_Beta; Fig. 1C). To advance this strategy, we bred high-carotenoid biparental families from genebank accessions (Fig. 1D); characterized gene action and allele effects (Fig. 2–6); developed KASP markers to tag variants in carotenoid genes and quantitative trait loci (QTL); and established trait-predictiveness of the markers in the biparental families (Fig. 5), diverse global germplasm (Fig. 6), and an African breeding program (Fig. 7). Thus, this study builds on previous work that primarily characterized carotenoid variation, by developing several of the resources needed to transfer high-carotenoid alleles from global genebanks into global breeding networks for sorghum provitamin A biofortification (Table 1–2).

**Table 1:** Origin of genebank accessions with yellow endosperm used for prebreeding

Accession ^a^	Family	Country of Origin ^b^	Botanical type ^b^	Previous Observations ^c^	
				Total Carotenoids (μg/g)	β-carotene (μg/g)
PI585348 (IS 24522)	1	Lebanon	Durra-caudatum	3.47	0.97
PI585369 (IS 24635)	1, 2	Lebanon	Kafir	3.29	0.96
PI569812 (IS 22366)	3, 4, 6	Sudan	Caudatum	3.23	0.91
PI562777 (IS 3686)	3, 5	United States	Durra	3.19	0.92
PI563430 (IS 10722)	5	United States	Guinea-durra	3.22	0.94
PI563447 (IS 10917)	4	United States	Guinea-durra	3.22	0.90
PI585365 (IS 24608)	6	Lebanon	Kafir	3.16	0.88
PI510917 (IS 22251)	2	Botswana	Guinea-caudatum	3.17	0.88

^a^ NPGS-GRIN plant inventory number, with the ICRISAT genebank ID in parentheses

^b^ Country of origin and botanical type from USDA GRIN database (USDA 2014)

^c^ Carotenoid concentrations data from a previous study (Cruet‐Burgos et al. 2020a) were used to select these accessions

**Table 2:** Single nucleotide polymorphisms (SNPs) used for KASP marker development and their corresponding genomic regions associated with carotenoid concentration

Marker ID	SNP ID ^a^	Alleles ^b^	Trait	A priori candidate gene	Distance to candidate gene	Genic region
snpSB00264	S6_46330663	C/**T**	Zeaxanthin	ZEP	385 kb	– ^**c**^
snpSB00265	S6_46717975	G/**A**	Zeaxanthin	ZEP	In gene	Intron
snpSB00266	S4_62459432	C/**T**	Zeaxanthin	MDS, ispF	46 kb	Intergenic
snpSB00267	S8_7569911	A/**T**	Zeaxanthin	(QTL)	–	Intergenic
snpSB00268	S10_57162947	C/**A**	Zeaxanthin	(QTL)	–	Intergenic
snpSB00276	S8_7570056	G/**T**	Zeaxanthin	(QTL)	–	Intergenic
snpSB00277	S6_47643430	C/**T**	Zeaxanthin	ZEP	921 kb	– ^d^
snpSB00279	S6_54257267	G/**A**	NA	β–OH	In gene	Intron
snpSB00280	S6_54257276	A/**G**	NA	β–OH	In gene	Intron
snpSB00281	S6_54257355	G/**T**	NA	β–OH	In gene	Intron
snpSB00282	S6_54257380	C/**G**	NA	β–OH	In gene	Intron

^a^ The SNP ID provides chromosome number (before the underscore) and the SNP position in v3.1.1 genome coordinates (after the underscore)

^b^ Alleles annotated as Ref/**Alt**, with the favorable high-carotenoid allele noted in bold

^c^ SNP lies within the 3’ UTR of a potassium channel gene (Sobic.006G093400)

^d^ SNP lies within the exon of a transmembrane transporter gene (Sobic.006G106700)

## Results

### Biparental progenies exhibit transgressive segregation for carotenoid content

To test the hypothesis that carotenoids can be increased in sorghum grain through breeding, the carotenoid content in six F_2:3_ biparental families and their parents was measured (Table 3). Lutein (Fig. 2) and zeaxanthin (Fig. 3) were detected in all the parental lines and progenies, whereas β-carotene, β-cryptoxanthin, and α-carotene were below the instrument detection limits. Lutein was the most abundant carotenoid across all samples. The average lutein concentration was 1.53 μg/g in the parental lines and 1.64 μg/g in the progenies, and the average zeaxanthin concentration was 1.25 μg/g in the parental lines and 2.08 μg/g in the progenies (Table 3).

**Table 3:** Concentration of carotenoids in parental lines and within F_2:3_ families

Family	Compound	Parents (μg/g)		F_2:3_ Progenies (μg/g)			
		PI585348	PI585369	Mean		Max	Min
Family 1	Lutein	2.6	1.9	2.1		6.4	0.3
	Zeaxanthin	1.7	1.4	1.5		3.6	0.4
	β-carotene	0.57	0.42	0.86		3.0	0.1
Family 2		PI510917	PI585369				
	Lutein	0.7	1.9	1.7		2.9	1.0
	Zeaxanthin	0.5	1.4	1.2		2.6	0.6
Family 3		PI562777	PI569812				
	Lutein	1.3	1.7	2.4		4.2	1.0
	Zeaxanthin	1.2	1.9	2.2		4.0	1.1
Family 4		PI563447	PI569812				
	Lutein	1.1	1.7	2.3		3.5	1.0
	Zeaxanthin	1.4	1.2	1.4		3.1	1.0
Family 5		PI563430	PI562777				
	Lutein	2.2	1.3	2.3		3.8	1.2
	Zeaxanthin	0.9	1.2	1.4		4.2	0.5
Family 6		PI585365	PI569812				
	Lutein	0.8	1.7	2.0		3.5	1.1
	Zeaxanthin	0.9	1.9	1.5		3.4	0.7
Average	Lutein	1.5		1.6			
	Zeaxanthin	1.3		2.1

Among the parents, PI585348 had the highest lutein concentration, and PI569812 had the highest zeaxanthin concentration, while PI510917 had the lowest concentrations of both carotenoids (Table 3). Within the families, Family 1 had the highest lutein concentration (Fig. 2A, Table 3) and Family 5 had the highest zeaxanthin concentration (Fig. 3D, Table 3). All families displayed transgressive segregation, with progeny concentrations both above and below parental values (Figs. 2 and 3). Family 1 displayed the widest range in lutein and zeaxanthin content (Figs. 2A and 3A), whereas Family 2 had the narrowest range (Figs. 2B and 3B). Family 3 had the highest increase in both lutein (59%) and zeaxanthin (39%) above the parental averages (mid-parent value), whereas family 1 had a decrease in both lutein (-7%) and zeaxanthin (− 3%). When tested using Method II (more sensitive to provitamin A carotenoids), family 1 (PI585348 ✕ PI585369) had higher concentrations of carotenoids: lutein ranged from 0.70 to 16.10 μg/g, zeaxanthin from 0.55 to 7.70 μg/g, and β-carotene from 0.10 to 3.00 μg/g (Fig. 4), compared to ranges of 0.3 to 6.4 μg/g for lutein and 0.4 to 4.2 μg/g for zeaxanthin obtained using Method I. Although the estimated concentrations were 1.8–2.5 times higher with method II, the results from the two methods were significantly correlated for both lutein (*r* = 0.46, *p* < 10^−12^) and zeaxanthin (*r* = 0.76, *p* < 10^−37^). Saponification has also been reported to show mixed results in lutein recovery across different foods (Irakli et al. 2011; Fitzpatrick et al. 2024). Together, these factors could have contributed to the lower correlation of lutein between the two methods. Method II was adopted for subsequent analysis of diverse germplasm due to its accuracy in reference samples and ability to detect β-carotene.

To compare the concentration and breeding potential of the biparental families, we assessed carotenoid levels in genotypes representing elite breeding germplasm used by national agricultural research systems in West Africa. Using HPLC Method II, we evaluated CSM-63, IRAT204, Macia, and Mota Maradi for their carotenoid content. These elite lines exhibited lower carotenoid concentrations and a narrower range than our selected parental lines, with lutein concentrations of 0.3 μg/g for Macia and IRAT204, 0.4 μg/g for Mota Maradi, and 0.6 μg/g for CSM-63 (Fig. 2). The zeaxanthin concentrations ranged from 0.5 μg/g for Macia, IRAT204, and CSM-63, whereas Mota Maradi had the highest content of 0.6 μg/g (Fig. 3). All four varieties had low β-carotene content, with CSM-63 having the highest concentration, of 0.2 μg/g, while the other three varieties just 0.1 μg/g (Fig. 4).

## KASP markers segregate and predict carotenoid content in biparental families

Eleven KASP markers were developed based on genomic regions associated with carotenoid concentration or genes involved in carotenoid biosynthesis (Table 2). Three markers (snpSB00268, snpSB00281, snpSB00282) were monomorphic among all the parent lines and progenies, so were discarded from subsequent analysis. For the remaining eight KASP markers, all three allelic classes were present among the parental lines and progenies (Online Resource Table S2). However, despite the presence of all the allelic classes, the progeny distributions were skewed towards a single genotype for each marker. Several markers were monomorphic within individual families and were excluded from further analysis. Families 4 and 5 were monomorphic at all loci whereas families 1, 2, 3, and 6 segregated at 3, 2, 5, and 6 loci respectively (Online Resource Table S2).

To test the hypothesis that MAS can be used as a selection method for sorghum grain carotenoids, an ANOVA and LSD test between carotenoid content in the F_2:3_ progenies across biparental families and genotype for eight KASP markers was performed. For lutein concentration obtained through Method I, seven markers were found to be significantly associated with concentration (*p* < 0.05) (Online Resource Table S4), and were able to distinguish between the lutein means of the genotype groups (Fig. 5). Three markers in proximity to QTLs for zeaxanthin (snpSB00265 [located within *ZEP*], snpSB00267, snpSB00276) could distinguish the means of the homozygous groups, but were unable to differentiate the heterozygous mean from the homozygous genotypes’ means (Figs. 5B, D, and E). Two markers in proximity to *ZEP* (snpSB00264, and snpSB00277) and two markers tagging *β–OH* (snpSB00279, snpSB00280) also had a significant association with lutein concentration (*p* < 0.05) and were able to distinguish between the homozygous genotype classes, but grouped the heterozygous classes with one of the homozygous classes (Figs. 5A, F, G, and H).

When analyzed at the individual family level, all the markers maintained significant association with lutein content (*p* < 0.05), however, the associations were family-specific (Online Resource Table S4). Markers snpSB00264 and snpSB00277 were significantly associated with lutein in families 3 and 6 (*p* < 0.05). In contrast, markers snpSB00267 and snpSB00276 exhibited significant associations in family 1, while snpSB00265, snpSB00279, and snpSB00280 were only associated with lutein in family 6 (*p* < 0.05). All eight markers were significantly associated with zeaxanthin concentration (*p* < 0.05; Fig. 5). Four markers (snpSB00264, snpSB00277, snpSB00279, and snpSB00280) had significant differences in zeaxanthin concentration and the means of the three genotype classes (*p* < 0.05) (Figs. 5A, F, G, and H)). Four of the markers (snpSB00265, snpSB00266, snpSB00267, and snpSB00276) grouped one homozygous class with the heterozygous class, which was differentiated from the second homozygous class (Figs. 5B–E). All the markers except snpSB00266 maintained a significant association with zeaxanthin content at the individual family level (Online Resource Table S4). Markers snpSB00267 and snpSB00276 had significant associations in family 1 (*p* < 0.05), while the rest of the markers exhibited significant associations in families 3 and 6 (*p* < 0.05).

To test the hypothesis that MAS can also be effective in selecting for β-carotene, we analyzed concentrations from biparental family 1 (PI585348 ✕ PI585369) obtained using Method II. For the β-carotene concentrations, two markers (snpSB00267 and snpSB00276) were significantly associated (*p* < 0.05; Figs. 5D–E). These two markers could differentiate the β-carotene concentrations between the homozygous groups, but grouped the heterozygous group with one of the homozygous groups.

## KASP markers also predict carotenoid content in diverse global germplasm

To test the hypothesis that these KASP markers are trait-predictive in global germplasm, nine of the markers were validated on a panel of genetically diverse inbred accessions (*n* = 90) representing a range of predicted carotenoid concentrations from a previous genomic prediction study (Cruet‐Burgos et al. 2020a). As expected for inbreds, the majority of the accessions carried the homozygous allele at the marker loci, while only 7% and 9% of accessions had heterozygous genotypes for marker snpSB00265 and snpSB00268, respectively (Online Resource Tables S3 and S8). Four of the markers (snpSB00264, snpSB00265, snpSB00267, snpSB00268) were skewed towards one allele while the rest were almost equally distributed.

Of the nine markers tested in the breeding families, six of them (snpSB00264, snpSB00265, snpSB00266, snpSB00267, snpSB00277, and snpSB00280) were significantly associated with lutein content (*p* < 0.05) and could differentiate between the means of the two homozygous classes (Fig. 6; Online Resource Table S5). Marker snpSB00265, with three allelic classes, could distinguish between the homozygous classes (AA and GG) but could not distinguish between the heterozygous (AG) and the homozygous class (AA). Five markers (snpSB00264, snpSB00265, snpSB00267, snpSB00277, snpSB00280) were significantly associated with lutein concentration (*p* < 0.05) in both the biparental families and the diverse germplasm (Figs. 5 and 6) and of these, two markers (snpSB00264 and snpSB00277) are the most promising for lutein selection because they were significantly associated (*p* < 0.05) in at least 2 biparental families in addition to the global germplasm.

All the markers tested were significantly associated with zeaxanthin concentration (*p* < 0.05) (Fig. 6; Online Resource Table S5), as observed for the biparental families, and could distinguish between the means of the homozygous groups. Only two markers (snpSB00265, snpSB00268) had three allelic classes present (Figs. 6B and E). Marker snpSB00265, as observed for lutein, clustered the phenotypic means of the AG and AA genotypes together. Marker snpSB00268 grouped the heterozygote mean with the homozygous groups’ mean. All the markers tested, except snpSB00282, were significantly associated with β-carotene concentration (*p* < 0.05), and could distinguish between the means of the homozygous groups (Fig. 6; Online Resource Table S5). Markers snpSB00265 and snpSB00268 showed a similar trend to that observed for lutein and zeaxanthin. Among the global diverse germplasm, snpSB00265 exhibited the most significant association with lutein (*p* < 10^−8^), zeaxanthin (*p* < 10^−20^), and β-carotene (*p* < 10^−8^) (Online Resource Table S5). Overall, for zeaxanthin, five markers (snpSB00264, snpSB00265, snpSB00277, snpSB00279, and snpSB00280) show potential for use in MAS because they were significantly associated with zeaxanthin concentration (*p* < 0.05) in the diverse germplasm and in at least two biparental families (Figs. 5 and 6: Online Resource Table S4 and S5).

## Inferred gene action at loci tagged by KASP markers associated with carotenoid content

To inform possible breeding strategies for carotenoid improvement, we used the biparental families to infer the putative gene action of the alleles at the loci under study (Table 2). Interestingly, among the alleles, different modes of gene actions were observed for lutein, zeaxanthin, and β-carotene concentrations. The alleles at marker (snpSB00265) exhibited additive action for lutein concentration, with the heterozygous mean being midway, and indistinguishable from both homozygous groups (Fig. 5B), while the alleles at two markers (snpSB00267, snpSB00276) exhibited a signal of dominance or a slight overdominance, with the heterozygous mean being higher than both homozygous means, but not statistically different from the homozygous group with the higher mean (Figs. 5D–E). The alleles at three markers (snpSB00265, snpSB00267, snpSB00276) exhibited positive partial dominance for zeaxanthin concentration, with the heterozygous class having an indistinguishable mean from the homozygous class with the higher zeaxanthin mean value (Figs. 5B, 6D, 6E).

The alleles of the rest of the markers (snpSB00264, snpSB00277, snpSB00279, snpSB00280) showed signals of negative partial dominance for lutein concentration, as evidenced by the heterozygous mean being indistinguishable from the homozygous group with the lower carotenoid mean (Figs. 5A, 5F–H), while exhibiting additive gene action for zeaxanthin concentration: the three genotypic classes were statistically different, with the heterozygous mean falling midway between the homozygous means (Figs. 5A, 6F–H). An additional marker that significantly associated with zeaxanthin concentration (*p* < 0.05), snpSB00266, had alleles that exhibited recessive or negative partial dominance action, where the heterozygous group mean was similar to the homozygous group with the lower zeaxanthin mean value. The alleles of two markers (snpSB00267, snpSB00276) significantly associated with variation in β-carotene concentration (*p* < 0.05) exhibited positive partial dominance (Figs. 5D–E).

## Evidence of marker segregation in a Senegalese pre-breeding and breeding program

In our work we mined alleles from genebank accessions (Previous study; summarized in Fig. 7A) and developed trait-predictive markers (This study; summarized in Fig. 7B). As a final test of the potential utility of the markers, we tested them in breeding and pre-breeding germplasm, at the Senegal national sorghum breeding program (Institut Sénégalais de Recherches Agricoles; ISRA (Fig. 7C), which has provitamin A content as a target trait in their product profiles (Online Resources Table S6). The germplasm consisted of elite germplasm (recently released varieties and elite breeding lines) and pre-breeding material (yellow donor lines and other donor lines being used for unrelated traits). The yellow donor lines were selected by the ISRA breeding program as potential donors for high-carotenoid alleles, based on grain color from the West African Sorghum Association Panel (Faye et al. 2021). The germplasm segregated at all markers, except snpSB00265 tagging *ZEP* and snpSB00281 tagging *β–OH* (Fig. 7C). Upon further analysis, marker snpSB00265 was found to perform poorly in this germplasm, with some genotypes expected to be harboring the favorable allele failing to amplify (Fig. 7D). New primers were designed on the antisense strand of this region, and the new marker, snpSB00869, had better performance. This marker will, in future screening, replace snpSB00265. Most of the germplasm was fixed for one homozygous class across all the markers (Online Resource Tables S9 and S10). The recently released varieties were fixed for the putative non-favorable alleles across all the markers, while the elite germplasm were mostly skewed towards the non-favorable alleles (Fig. 7C). The donor lines with yellow-colored grain, a phenotype we have previously shown to be significantly correlated with grain carotenoid content (*p* < 0.05) (McDowell et al. 2024) had the highest frequency of favorable alleles across the markers tested (Fig. 7C). By contrast, the other donor lines, used for unrelated traits, had a moderate frequency of the favorable alleles (Online Resource Table S10).

## Discussion

### Transgressive segregation in carotenoid content offers potential for biofortification

Biofortification breeding is a key approach to increasing provitamin A concentrations, but prebred high-carotenoid germplasm and trait-predictive markers for sorghum breeding for carotenoids have not yet been available to the breeding community (Cruet‐Burgos et al. 2020a). In this study, six biparental families were developed from selected lines with varying carotenoid concentrations. Importantly, progeny in all the families exhibited transgressive segregation, with carotenoid concentrations both higher and lower than their respective parental genotypes (Figs. 2–4). This result can be explained by either of two competing hypotheses: (1) the observed segregation may be due to measurement error, or (2) the parents contributed complementary alleles at different loci, leading to additive or interactive genetic effects. Similar patterns of transgressive segregation have been reported in other breeding efforts, including for β-carotene concentration in segregating F_2_ families (Worzella et al. 1965) and in fixed lines such as RILs (Fernandez et al. 2008).

Taken together, the segregation of alleles within the parental lines (Online Resource Table S7) and the transgressive segregation of the progeny (Figs. 2–4) support the hypothesis that the parents contribute complementary favorable alleles at multiple loci. This is the more favorable hypothesis for carotenoid biofortification, as it suggests that sustained genetic gain and therefore higher carotenoid concentrations may be achievable through forward breeding. Although the biparental breeding families have higher concentrations than the current elite germplasm in West Africa (Figs. 2–4), these lines were closed-pollinated to maintain the genetics, and this practice has been previously linked to altered accumulation of carotenoids (Kean et al. 2011). Kean et al 2011 reported an increase in carotenoids of 8–184% in bagged panicles when compared to their unbagged controls, suggesting a varietal response. A comparative study of open vs closed-pollination is thus warranted, to clarify the genotypic effects of bagging on carotenoid content. The highest β-carotene concentration observed among the progenies was 3 µg/g, reaching 25% of the minimum target biofortification value of 12 µg/g (Fig. 4) and 6% of the EAR. This highlights that, while marker-assisted backcrossing could be a valuable starting point for trait introgression, it might not be sufficient on its own to reach target provitamin A levels. Achieving those levels may require a recurrent selection breeding scheme to progressively accumulate favorable alleles, perhaps leveraging epistasis (Fig. 2–4) to harness *selection-induced genetic variation* (Carlborg et al. 2006). To that end, it is important to identify and cross the highest-carotenoid lines from both our biparental families and previously identified germplasm (Fernandez et al. 2008; Cruet-Burgos and Rhodes 2023). Developing biofortified lines that approach or surpass target values would directly benefit consumers in vitamin A-deficient regions by providing a locally adapted, climate-resilient, and culturally acceptable dietary source of provitamin A—particularly critical when other crops fail due to extreme weather events. Future studies could also directly mine useful alleles for known carotenoid-related genes from global genebanks using pangenome sequences of diverse accessions and introgression line resources that move genebank alleles into elite backgrounds. Also, given the presence of antinutritional compounds such as tannins in sorghum, work is needed to assess the bioavailability of provitamin A carotenoids in these improved lines to ensure that increased carotenoid levels provide real nutritional benefits.

## Breeding implications of inferred gene action in carotenoid candidate genes

Understanding the gene action underlying phenotypic variation can guide prioritization of variety types (hybrid, open-pollinated, or pureline) (Fasoula and Fasoula 2010) and inform breeding strategy. Deviation of progeny means from midparent value is a useful initial indicator for general gene action (additive vs. dominance) (Bernardo 2020). In F_2_ or backcross families, substantial deviation from the midparent value suggests epistatic interactions (Carlborg et al. 2006). In this study, the biparental F_2:3_ families had a carotenoid mean value above the midparent value, with most families (4 of 6) showing substantial position deviations from the midparent value (Figs. 2–4), suggesting epistatic interactions among the QTL. In maize, epistasis plays a minor role in determining carotenoid traits: 14 interactions with minimal effect were observed across nested association mapping (NAM) families (Diepenbrock et al. 2021), another study of 10 NAM families found no interactions (Chandler et al. 2013). Modeling interactions across the carotenoid pathway (Fig. 1A) could clarify the genetic architecture underlying carotenoid accumulation in sorghum and navigate the complexity (Figs. 1B–C).

Overall, across all markers evaluated, two primary forms of gene action were observed: (i) additive, in which one homozygous genotype was consistently associated with higher carotenoid concentrations, the alternative homozygous with lower concentrations, and the heterozygotes displayed intermediate concentrations; and (ii) dominance, in which one homozygous genotype and the heterozygotes were associated with a higher carotenoid concentration relative to the alternative homozygote (Fig. 5). The additive intralocus effect is consistent with previous reports in other cereals such as maize, rice, and wheat (Harjes et al. 2008; Yan et al. 2010; Babu et al. 2013; Colasuonno et al. 2017), whereas Kandianis et al. (Kandianis et al. 2013) suggested that variation in maize *β-*carotene was better explained by models that include both dominance and additive effects. Markers tagging key carotenoid biosynthesis genes (*β–OH* and *lycE*) have been reported to have largely additive effects in maize and have been successfully used in marker-assisted breeding for elevated provitamin A by stacking the favorable alleles (Saltzman et al. 2013; Dhliwayo et al. 2014). This strategy has enabled the release of maize varieties accumulating 40–70% more provitamin A than the targeted 15 μg/g (Bouis and Saltzman 2017), as well as prebreeding lines with even higher concentrations (Menkir et al. 2015).Thus, the use of MAS to develop inbred lines that fix the favorable alleles showing additive effects could be an efficient strategy for increasing the carotenoid content in sorghum.

## Prospects for deploying trait-predictive markers in global breeding networks

In this study, we developed 11 markers, at least 9 of which were validated as trait-predictive KASP markers, and these are publicly available through Intertek for deployment across breeding programs. Validation of KASP markers across diverse germplasm, other than the family where the markers were developed, ensures that markers are robust, broadly applicable, and consistently predictive for the target trait (Table 2, Figs. 5 and 6). KASP markers target specific alleles at a loci, thus might miss most rare alleles or common alleles not represented in the family used for marker development (Cobb et al. 2019; Marla et al. 2023). The most promising markers for lutein, zeaxanthin and β-carotene from the biparental family analyses were also predictive in diverse global germplasm (compare Figs. 5 and 6). Five markers (snpSB00264, snpSB00265, snpSB00277, snpSB00279, snpSB00280) demonstrated potential for use in selecting for zeaxanthin content across breeding families and diverse germplasm, while two of these (snpSB00264 and snpSB00277) also showed potential for lutein selection (Figs. 5 and 6).

The markers in common for both lutein and zeaxanthin suggest a pleiotropic effect and provide a valuable opportunity for the simultaneous selection for both traits, especially in the early biofortification stages when the Max_Car breeding strategy is employed (Fig. 1B). However, the partial overlap between some of the markers might mean that the tagged genes control flux into different metabolic branches (α or β), thus some markers will show a stronger association with only lutein or only zeaxanthin, depending on which branch the tagged gene is active. Such pathway positional dependency has been previously reported (Harjes et al. 2008; Kandianis et al. 2013; Owens et al. 2014), in which overlapping QTL are more frequently detected for carotenoid molecules within the same biosynthetic branch compared to those in different branches. This potentially explains why marker snpSB00265 is more strongly associated with zeaxanthin than lutein (Figs. 5 and 6). The marker lies inside the coding region of *ZEP* (Fig. 7C and Table 2), which encodes a β-branch enzyme consistently associated with zeaxanthin in sorghum and maize (Owens et al. 2014; Suwarno et al. 2015; Cruet‐Burgos et al. 2020a). Achieving co-enhancement of lutein and zeaxanthin in the Max_Car strategy will require stacking of multiple favorable alleles across the pathway rather than relying on just a few common loci.

For the β-carotene content, only two markers were common between biparental family 1 and the diverse germplasm (snpSB00267 and snpSB00276) (Fig. 4). The predictive ability of these markers requires further investigation in additional biparental families. However, zeaxanthin and β-carotene are both in the β-branch of the carotenoid biosynthetic pathway (Fig. 1A), and their concentrations are highly correlated. This was evident in family 1 (*r* = 0.69, *p* < 10^−15^) (Online Resource Table S1) and the global germplasm (*r* = 0.83, *p* < 10^−15^) (Online Resource Table S8) and is consistent with prior findings in other diverse germplasm (*r* = 0.83–0.85, *p* < 10^–3^) (Cruet‐Burgos et al. 2020a; McDowell et al. 2024). It thus can be hypothesized that the zeaxanthin-predictive markers can be used across breeding families to effectively select for both zeaxanthin and β-carotene concentrations (Fig. 1B–C). By contrast, we hypothesize that implementing the Max_Beta breeding strategy (Fig. 1C) will enhance the accumulation of the provitamin A compounds, specifically, β-carotene and β-cryptoxanthin, by accumulating favorable alleles that reduce their degradation into downstream compounds, including zeaxanthin. This targeted reduction in provitamin A degradation will not only increase the provitamin A content but might alter the metabolic flux within the pathway, potentially shifting the currently observed correlations. If this hypothesis holds true, then there will be a need to develop distinct markers for β-carotene and zeaxanthin to enable independent selection.

Two other markers (snpSB00264 and snpSB00277; Figs. 5 and 6) that show potential for global deployment in selecting for all the carotenoids tested in this study are in proximity to *ZEP* (385 kb and 921 kb, respectively; Fig. 7B and Table 2). This suggests potential linkage disequilibrium between the markers and *ZEP*, allowing their use as indirect markers (Online Resource Table S4–5; Figs. 5 and 6), however leveraging pangenomic resources to uncover variation in structural genes involved in carotenoid biosynthesis could facilitate the identification of causal variants and the development of associated KASP markers. Lastly, of the four markers designed within the *β–OH* gene, two (snpSB00279, snpSB00280), are good candidates for MAS given their predictiveness for lutein, zeaxanthin, and β-carotene variation (Online Resource Table S4 and S5: Figs. 5 and 6). This observation is consistent with the role of *β–OH* in catalyzing the β-ring hydroxylation of β-carotene to produce β-cryptoxanthin and zeaxanthin (Fig. 1). Further, the maize homolog of the *β–OH* gene has been successfully targeted for MAS in maize biofortification (Babu et al. 2013; Muthusamy et al. 2014; Goswami et al. 2019). Together, these findings highlight the potential of leveraging orthologous gene function and observed trait associations to accelerate MAS in carotenoid breeding.

## Conclusions

This study demonstrates that MAS for provitamin A biofortification in sorghum with alleles mined from genebanks (Fig. 1) is likely to be feasible (Figs. 2–7). Transgressive segregation of grain carotenoid concentrations was observed across six F_2:3_ biparental families (Figs. 2–4), providing evidence for breeding as a tool for increasing carotenoid concentrations in sorghum grain. Two of the 11 KASP markers developed in this study predict β-carotene content in both the global germplasm and the biparental family evaluated, and require further validation. Five markers predict zeaxanthin content in multiple biparental families and global germplasm and have potential for use as indirect markers for provitamin A carotenoids. Deployment of these markers in an active Senegalese breeding program revealed that the favorable alleles for high carotenoid accumulation were predominantly present in the yellow-grain donor lines, whereas they were mostly absent in elite germplasm and newly released varieties derived from non-yellow donor backgrounds (Fig. 7C). This suggests that this breeding program has sufficient genetic variation that can be utilized for carotenoid biofortification. Together, these findings advance sorghum biofortification efforts to alleviate vitamin A deficiencies in drought-prone regions of the developing world.

## Methods

### Development of biparental families

Eight sorghum inbred accessions with medium to high carotenoid concentrations (PI585348, PI585369, PI569812, PI562777, PI563430, PI563447, PI585365, PI510917) (Cruet‐Burgos et al. 2020a) were used as parents to develop six biparental F_2:3_ families (Fig. 1D, Table 1). The initial crosses were developed using the plastic bag emasculation method (Laxman 1997). F_1_ plants were grown in a greenhouse, and leaf tissue from 2-week-old plants was collected and sent to Intertek AgriTech (Alnarp, Sweden) for DNA isolation and KASP marker testing to identify true hybrids. The true hybrids were then self-pollinated. The F_2_ progeny of the PI585348 ✕ PI585369 cross was grown at Kansas State University Agronomy North Farm (39.206, − 96.593) during Summer 2019, and the remaining F_2_ progeny were grown during Summer 2020. Leaf tissue from 2-week-old F_2_ plants was collected and sent to Intertek AgriTech (Alnarp, Sweden) for KASP marker testing. The F_2_ plants were self-pollinated and the resulting F_2:3_ grain was harvested and stored at − 80 ℃ until carotenoid extraction. The percentage deviation (*D*) of the progeny mean from midparent value was calculated for each family:

$$D = \left( {{{\left( {family{ }mean{ } - midparent{ }value} \right)} \mathord{\left/ {\vphantom {{\left( {family{ }mean{ } - midparent{ }value} \right)} {midparent{ }value}}} \right. \kern-0pt} {midparent{ }value}} \times { }100} \right).$$

## Development of KASP Markers

Single nucleotide polymorphisms (SNPs) in genomic regions previously identified in genome-wide association studies to be significantly associated with carotenoid concentrations (Cruet‐Burgos et al. 2020a) or in carotenoid biosynthesis genes were used to develop eleven KASP markers (Table 2; Fig. 7B): seven SNPs previously associated with zeaxanthin concentration (S4_62459432, S6_46330663, S6_46717975, S6_47643430, S8_7569911, S8_7570056, and S10_57162947) and four novel SNPs (S6_54257267, S6_54257276, S6_54257355, and S6_54257380) within the coding sequence of Sobic.006G188200, a putative *β-carotene 3-hydroxylase* (*β–OH*) that underlies carotenoid variation in maize (Yan et al. 2010). The SNPs, along with 100 bp flanking sequences, were obtained from the *Sorghum bicolor* reference genome (v3.1.1) available through Phytozome at https://phytozome-next.jgi.doe.gov/info/Sbicolor_v3_1_1. These sequences were submitted to Intertek AgriTech (Alnarp, Sweden) for KASP marker development.

## Extraction and Quantification of Carotenoids

An initial method (Method I) was optimized for the extraction and quantification of lutein and zeaxanthin, which are the carotenoids present in the highest concentrations in sorghum grain. Carotenoid extractions from grain of the F_2:3_ and parental lines were carried out under yellow light to avoid photodegradation, using a modified solid phase saponification method (Irakli et al. 2011). The six biparental families were considered biological replicates for analysis. For each line *(n* = 930), about 2 g of grain derived from one panicle were ground for analysis. Resolution of lutein and zeaxanthin was conducted using a Perkin Elmer LC 300 UHPLC (Waltham, Massachusetts), attached to a PDA. A 4 μL aliquot of extract was injected into a Zorbax SB-CN column (2.1 × 100 mm 3.5um, Agilent Technologies, California, U.S.). Refer to Online Resource File S1 for additional details on the extraction and HPLC method. Method I failed to detect β-carotene, the provitamin A carotenoid present in the highest concentrations in the grain, and this necessitated the development of a second method (Method II) optimized for the extraction and quantification of β-carotene in addition to lutein and zeaxanthin.

Because β-carotene is undetectable in low-carotenoid sorghum lines, and HPLC is costly and low-throughput, Method II was applied only to family 1 (PI585348 ✕ PI585369), which exhibited the highest total carotenoids using Method I. Carotenoid extractions from grain of 249 F_2:3_ lines from family 1 were carried out under yellow light. About 2 g of grain from one panicle per line were ground together for analysis of the individual lines and three replicates per ground panicle were analyzed for carotenoid content. The carotenoids were quantified using HPLC Flexar (PerkinElmer, United States), attached to a PDA. Briefly, a 7 μL aliquot of sample was injected through a C30 column (150 × 2 mm I.D. S-3 µm; YMC American, Inc.) at 35 °C for the carotenoid separation. Refer to Online Resource File S1 for additional details on the extraction and HPLC method. The correlation between lutein and zeaxanthin concentrations measured by Methods I and II was confirmed. Method II was adopted for subsequent analyses due to its accuracy (determined by measurement of a reference corn sample) and ability to additionally detect β-carotene. Total carotenoids were calculated as the sum of lutein, zeaxanthin, and β-carotene concentrations (where detected).

## Statistical test of trait predictiveness of markers

Predictive ability of the polymorphic KASP markers (Table 2) was assessed using analysis of variance (ANOVA) in RStudio ver2023.6.1.524 (Posit team 2023) with the ‘aov’ function. Lutein and zeaxanthin concentrations in the F_2:3_ progenies of all six biparental families, as well as β-carotene concentrations in family 1 (PI585348 ✕ PI585369), were independently tested for each of eight polymorphic markers. In the global germplasm, lutein, zeaxanthin and β-carotene concentrations were independently tested for each of nine polymorphic markers. Markers with an association of *p* < 0.05 were considered statistically significant. Differences in the means of genotype classes for each marker were tested using a least significant difference (LSD), implemented via the ‘LSD.test’ function in the agricolae R package.

## Validation of markers in diverse germplasm and breeding lines

To validate the trait predictiveness of the KASP markers, a panel of 134 diverse global accessions sourced from USDA-GRIN were analyzed. The accessions, primarily from Nigeria, Niger, Lebanon, Ethiopia, Sudan, USA, and Botswana, were selected based on their genomic best linear unbiased prediction (gBLUPs) (Cruet‐Burgos et al. 2020a), representing the top 5%, bottom 5%, and middle-ranking accessions to capture a wide range of the potential allelic variation. Briefly, the accessions were grown under controlled conditions in a greenhouse, and leaf tissue was collected 2 weeks after germination and sent to Intertek Agritech (Alnarp, Sweden) for DNA isolation and KASP genotyping with 9 of the 11 markers that were classified as “good” or “very good” based on the quality of scoring and call rate in the biparental families (snpSB00264, snpSB00265, snpSB00266, snpSB00267, snpSB00268, snpSB00277, snpSB00279, snpSB00280, snpSB00282). From the initial 134 accessions grown, only 90 developed sufficient grain for analyses. The grain was harvested 42 days after flowering and lutein, zeaxanthin, and β-carotene concentrations were determined using Method II. For each accession, two biological replicates were included for analysis, each with three technical replicates. The trait predictiveness of the markers was determined as described for the biparental families. The markers were additionally tested in a panel of recently released varieties, elite breeding lines, and improved varieties (*n* = 136) selected from the Senegalese national sorghum breeding program at Institut Sénégalais de Recherches Agricoles (ISRA). This selection included four genotypes representing elite breeding germplasm used by national agricultural research systems in West Africa: PI655981 (CSM-63), PI656031 (IRAT204), PI565121 (Macia), and PI656050 (Mota Maradi) (Faye et al. 2022).

## Supplementary Information

Below is the link to the electronic supplementary material.

Supplementary file1 (DOCX 5035 KB)

Supplementary file2 (XLSX 450 KB)

## Data Availability

The data generated and/or analyzed in this study are available in this research article. The detailed methods for carotenoid extraction and determination via HPLC, carotenoid data, and KASP genotype data have been provided as supplementary materials. The carotenoid concentrations and genotype data for the Zeaxanthin GWAS (Fig. 7A) was previously published by (Cruet‐Burgos et al. 2020b).

## Code availability

All codes utilized in this article are publicly available on GitHub: https://github.com/Linly-BN/CSU_RhodesLab/tree/main
