# Supplementary material for: Advancing provitamin A biofortification in sorghum: from allele mining and marker development to prebreeding and breeding
Source: Theor Appl Genet. 2026 Apr 27;139(5):136. doi: 10.1007/s00122-026-05245-2 (PMC13121180; doi:10.1007/s00122-026-05245-2)
Supplement: Supplementary file 1 — Supplementary file1 (DOCX 5035 KB) [file 122_2026_5245_MOESM1_ESM.docx]

## **ONLINE RESOURCE**

**Supplementary File 1**

**Advancing provitamin A biofortification in sorghum: From allele mining and marker development to prebreeding and breeding**

Clara Cruet-Burgos^1,2#^, Linly Banda^1#^, Jacques M. Faye^3^, Cyril Diatta^4^, David Zapata^1^, Geoffrey P. Morris^2^, and Davina H. Rhodes^1^*

^1^Department of Horticulture & Landscape Architecture, Colorado State University, Fort Collins, CO 80523, USA

^2^Department of Soil & Crop Science, Colorado State University, Fort Collins, CO 80523, USA

^3^ Institut Sénégalais de Recherches Agricoles, Centre d’Étude Régional pour l'Amélioration de l'Adaptation à la Sécheresse, Thiès, Sénégal and Centro Internacional de Mejoramiento de Maíz y Trigo (CIMMYT)

^4^ Institut Sénégalais de Recherches Agricoles, Centre d’Étude Régional pour l'Amélioration de l'Adaptation à la Sécheresse, Centre National de Recherches Agronomiques, Bambey, Sénégal

^#^ Equal contributions

*Corresponding author: [davina.rhodes@colostate.edu](mailto:davina.rhodes@colostate.edu), ORCID 0000-0003-2140-1519

##

## ***Extraction and Quantification of Provitamin A carotenoids - Method I****

Briefly, 20 mg of the sorghum flour was obtained from grinding approximately five grains using a Bead Ruptor Elite (Omni International, Kennesaw, GA). Flour was transferred to a 1.5 mL tube along with 20 mg of ascorbic acid and 400 μL of 1 mg/mL of butylated hydroxytoluene (BHT) in ethanol. Samples were vortexed for 1 minute and incubated for 5 minutes in a water bath at 80 °C. Next, 20 μL of a solution of potassium hydroxide (80% w/v, in water) was added and the tubes vortexed for 1 minute. Samples were incubated again at 80 °C for 15 minutes, with mixing every 5 minutes. Following incubation, the samples were brought to room temperature and centrifuged for 5 minutes at 1900 x g. The supernatant was transferred to a new 1.5 mL tube and an additional 400 μL of absolute ethanol with 1 mg/mL of BHT was added to the residue. The tube with residue was then vortexed for 1 minute and centrifuged for 5 minutes at 1900 x g. After centrifugation, the supernatant was combined with the previous extract, vortexed for 30 seconds and centrifuged for 5 minutes at 5000 x g. The combined supernatant was then transferred to a new 1.5mL Eppendorf tube and evaporated to dryness under a gentle N_2_ stream at room temperature. Finally, the residue was reconstituted in 100 μL of Ethanol:Ethyl Acetate (1:1 v/v) and centrifuged for 5 minutes at 5000 x g. An aliquot of 40 μL of the clear supernatant was utilized for the HPLC analysis.

Resolution of lutein and zeaxanthin was conducted using a Perkin Elmer LC 300 UHPLC (Waltham, Massachusetts), attached to a photodiode array detector (PDA). A 4 μL aliquot of extract was injected into a Zorbax SB-CN column (2.1x100mm 3.5um, Agilent Technologies, California, U.S‎.). Mobile phase A consisted of water:methanol, 1:1 v/v, and mobile phase B consisted of a 0.1% formic acid in acetonitrile. Carotenoids were resolved using a low of 0.4 mL/minute and the following gradient: 55 % A and 45 % B for 0.5 min, 15.6 % A and 84.4 % B for 5.5 minutes, 5 % A and 95 % B for 0.2 minute, and a final 6.8 minutes of 55 % A and 45 % B making a total 13 minutes. The carotenoids were detected at 445 nm by applying six-point standard curves for β-carotene, β-cryptoxanthin, α-carotene, zeaxanthin, and lutein ranging from 0.063 to 2 µg/mL. Carotenoid concentrations were then calculated using a linear regression *y* = *mx* + *b*, where *y* = concentration and *x* = area of the six-point standard curve/areas of IS. The regression equation and correlation coefficient (*R*^2^) were obtained.

***Extraction and Quantification of Provitamin A carotenoids - Method II****

Method II was optimized for the extraction and quantification of provitamin A carotenoids, which are present in very low concentrations in sorghum grain, making them more difficult to detect. β-carotene is the provitamin A carotenoid present in the highest concentrations in the grain. Since β-carotene is undetectable in low carotenoid sorghum lines, and due to the high cost and low-throughput of HPLC, β-carotene was only measured in the family with the highest total carotenoids, identified using Method I. In addition to β-carotene, lutein and zeaxanthin were also measured with Method II, to confirm correlation between Method I and Method II. Carotenoid extractions from grain of 249 F_2:3_ lines from family 1 (PI585348 ✕ PI585369) were carried out under yellow light. Seeds from one panicle per line were ground together for analysis of the individual lines and three replicates per ground panicle were analyzed for carotenoid content.

Briefly, frozen grain samples were ground into powder using a stainless steel analytical mill (IKA A11 basic), and 300 ± 0.05 mg of flour was weighed in a 2 mL screw-cap microtube. The extraction solvent containing 0.1 μg/mL of retinyl palmitate (internal standard) and 0.01% butylated hydroxytoluene (BHT) in ethyl acetate: acetone: ethanol (1:1:1, v/v/v), and two stainless steel beads were added to the micro tube. The tubes were placed into a homogenizer (Omni Bead Ruptor 24 Elite) for 2 cycles of 2.5 minutes at 2.6 m/s, with a brief pause of 25 seconds in between. The samples were then centrifuged for 4 minutes at 18213 x g, and the supernatant was transferred into a 1.5 mL Eppendorf tube. The extraction was repeated from the pellet with 700 μL of methyl tert-butyl ether (MTBE). The combined supernatant was mixed, centrifuged for 30 seconds at 18213 x g, and transferred into a new 2 mL screw-cap self-standing microtube. The samples were concentrated to dryness under a gentle stream of N_2_ at room temperature. The residues were reconstituted in 250 μL of ethyl acetate:ethanol (50:50 v/v), vortexed, and centrifuged for 30 seconds at 18213 x g. An 80 μL aliquot of the extract was transferred into an insert housed in an amber HPLC vial.

The carotenoids were quantified using HPLC Flexar (PerkinElmer, United States), attached to a photodiode array detector (PDA). Briefly, a 7 μL aliquot of sample was injected through a C30 column (150 x 2 mm I.D. S-3 µm; YMC American, Inc.) at 35 °C for the carotenoid separation. The autosampler was maintained at 7 °C. Mobile phase A consisted of 95% methanol: 1.0 M ammonium acetate (98:2 v/v), and mobile phase B of MTBE, methanol, and 1.5% ammonium acetate (90:8:2, v/v/v). Carotenoids were resolved using a flow of 0.48 mL/minute with the following gradient: 100 % A for 3 minutes, 20 % A and 80 % B for 1.5 minutes, 100 % B for 2.5 minutes, back to 100 % A for the final 6 minutes, making a total 13 minutes. The internal standard was quantified at 325 nm, and carotenoids were detected at 450nm. Quantification was performed using TotalChrom Navigator Version 6.3.4. Samples and standard data were normalized, and the concentrations were calculated using the calibration curve of the corresponding standard as a reference. Five-point external standard curves were constructed from the standard mix ranging from 0.25 to 5 μg/mLfor lutein, zeaxanthin, β-cryptoxanthin, and α-carotene, whereas from 0.1 to 2.5 μg/mL for β-carotene. Carotenoid concentrations were determined by linear regression (*y* = *mx* + *b)*, where *y* is the concentration and *x* is the peak area relative to the internal standard, using the regression equation and correlation coefficient (*R*^2^) of each five-point curve.

* A reference sample of corn grits was measured with both methods to determine the accuracy, Method 1 detected 3.5 - 4.9 μg/g of lutein and 5.0 - 8.0 μg/g of zeaxanthin while method 2 detected 7.8 - 8.3 μg/g lutein, 10.2 - 10.6 μg/g zeaxanthin, and 0.9 μg/g of 𝛽-carotene, making a total carotenoid content of 19.8 against a known range of 20 - 25 μg/g. It was thus concluded that Method II was more accurate.
